# Advances and challenges in anti-cancer vaccines for multiple myeloma

**DOI:** 10.3389/fimmu.2024.1411352

**Published:** 2024-08-01

**Authors:** Pegah Abdollahi, Hanne Marie Norseth, Fredrik Schjesvold

**Affiliations:** Oslo Myeloma Center, Department of Hematology, Oslo University Hospital, Oslo, Norway

**Keywords:** multiple myeloma (MM), immunotherapy, vaccine, tumor-associated antigen (TAA), tumor-specific antigen (TSA), idiotype (Id)

## Abstract

Multiple myeloma (MM) is a hematological cancer marked by plasma cell accumulation in the bone marrow. Despite treatment advancements, MM remains incurable in most patients. MM-associated immune dysregulation fosters disease progression, prompting research into immunotherapy to combat the disease. An area of immunotherapy investigation is the design of myeloma vaccine therapy to reverse tumor-associated immune suppression and elicit tumor-specific immune responses to effectively target MM cells. This article reviews vaccine immunotherapy for MM, categorizing findings by antigen type and delivery method. Antigens include idiotype (Id), tumor-associated (TAA), tumor-specific (TSA), and whole tumor lysate. Myeloma vaccination has so far shown limited clinical efficacy. However, further studies are essential to optimize various aspects, including antigen and patient selection, vaccine timing and sequencing, and rational combinations with emerging MM treatments.

## 1 Introduction

Multiple myeloma (MM) is a hematological malignancy characterized by the accumulation of clonal plasma cells in the bone marrow (BM) and secretion of monoclonal immunoglobulins, leading to the hallmark symptoms known as CRAB (hypercalcemia, renal insufficiency, anemia, and bone lesions). Despite the advent of numerous new treatments that have significantly improved outcomes, MM remains largely incurable, and the disease continues to have a fatal outcome for most patients in advanced stages (1). Increasing research attention have been placed on immunotherapy that targets MM cells utilizing the host immune system to eliminate the malignant cells in the BM (2). Myeloma is associated with immune dysregulation due to ineffective antigen presentation and effector cell dysfunction creating an immunosuppressive milieu that fosters disease progression. An area of investigation is the design of myeloma vaccine therapy (2). Cancer vaccination is a therapeutic approach designed to activate the immune system to recognize and combat cancer cells. These vaccines can serve as preventive measures, known as prophylactic vaccines, or as treatment options for individuals already diagnosed with cancer, referred to as therapeutic vaccines (3). The choice of antigen targeted by any cancer vaccine is critical to its clinical efficacy. Tumor antigens are classified into two broad categories: tumor-associated antigens (TAAs) and tumor-specific antigens (TSAs) (4). TAAs are self-antigens that are either preferentially or abnormally expressed in tumor cells but may also be present at some level in normal cells. TSAs, also known as neoantigens, include antigens that are encoded solely by cancer cells and are tumor-specific, eliciting high-affinity T cell response (5). Vaccines are categorized into shared and personalized vaccines. Shared antigens are public antigens and can be presented by a relatively common human leukocyte antigen (HLA) allele in patients. Such vaccines are promising candidates for off-the-shelf immunotherapy. On the other hand, personalized cancer vaccines have recently gained more attention due to advancements in high-throughput gene sequencing, mass spectrometry (MS) and bioinformatics, which enable the identification of HLA-bound peptides and the prediction of unique personalized neoantigens (6). In addition, cancer vaccines can be delivered through various platforms such as peptide vaccines, RNA vaccines, DNA vaccines, viral vaccines, and antigen-presenting cell (APC)/dendritic cell (DC) vaccines (7). Today, most vaccines under clinical investigation involve the delivery of tumor antigens in combination with an adjuvant or other costimulatory factors. Adjuvants are essential components of cancer vaccines as they enhance immune responses by activating innate immune pathways. Various adjuvants, such as Toll-like receptor (TLR) agonists and cytokines are used to improve the efficacy of cancer vaccines (3). For more detailed information on cancer vaccines, please refer to references 3-7.

This article aims to review major findings in vaccine immunotherapy against MM, categorized based on the type of antigens and means of their delivery either as peptide-, protein-, DNA- or DC-based vaccines. The discussed tumor antigens encompass idiotype (Id) antigens, TAA, TSA, and whole tumor lysate. In MM, malignant plasma cells secrete a monoclonal immunoglobulin (paraprotein) containing tumor-specific antigenic determinants known as Id. Ids are formed through gene rearrangement during B-cell maturation and somatic hypermutation (8). Although Id antigens are a subset of TSA, a dedicated section to these antigens is included in this review due to the historical significance of investigating vaccines against myeloma Id antigens.

## 2 Idiotype vaccine

### 2.1 Id protein/peptide- or DNA-based vaccine

One of the initial investigations of Id-vaccines goes back to a study in 1995 by Kwak et al., where they immunized a healthy sibling BM donor with myeloma immunoglobulin from the plasma of the recipient. Detection of a lymphoproliferative response, with recovery of a recipient CD4+ T-cell line with unique specificity for myeloma Id, was a proof of concept that immunization with Id may represent a new strategy for enhancing the specific antitumor effect (9). Later, more studies investigated the potential of Id vaccination as a treatment strategy (10, 11). A cohort of five patients with stage I-III MM underwent repeated immunization with autologous serum M-component, administered in alum as an adjuvant. While successful in three patients, the elicited immune response was modest in magnitude and short-lived (10). Subsequent studies introduced immunogenic carrier proteins such as keyhole limpet hemocyanin (KLH) (12) or filamentous phage (13) with or without adjuvants such as granulocyte-macrophage colony-stimulating factor (GM-CSF) (12, 14, 15), interleukin (IL)-2 (12), IL-12 (14) to enhance anti-tumor immunity of Id proteins. Even though these strategies were capable of evoking tumor-specific immune responses, the clinical response was modest (11, 15). For instance, in the study by Osterborg et al., Id vaccine was used together with GM-CSF in five stage II MM patients. All patients developed an Id-specific T-cell immunity, but a significant reduction in M-component concentration was noted in only one individual (15). Massaia et al., vaccinated patients with minimal residual disease (MRD) following high-dose chemotherapy (HDT) using the Id protein with KLH and a low dose of the adjuvant cytokines GM-CSF or IL-2. An idiotype-specific T-cell response was documented in 75% of patients with no decrease in tumor burden (12). Another study explored the utilization of Id-specific protein conjugated to KLH with low doses of GM-CSF as maintenance therapy in the first remission post-HDT and peripheral blood progenitor cell infusion. A retrospective case-matched analysis revealed similar survival and progression free survival (PFS) durations between the vaccine and control groups who were treated with interferon (IFN)-*α* and/or dexamethasone as maintenance therapy (16).

More efforts were employed where Id DNA-based vaccine encoding patient-specific single chain variable fragment, or Id linked to fragment C (FrC) of tetanus toxin was investigated in a non-randomized phase I clinical trial. Fourteen patients received vaccine initiated at least 6 months post-autologous stem cell transplant (ASCT)/HDT. Over the 52-week study period, serum paraprotein was undetectable, decreased or remained stable for ten patients (71%), whilst ongoing complete response (CR)/partial response (PR) was maintained for 11 patients (79%). Moreover, median time to progression (TTP) was 38.0 months for 13 patients. However, due to prior ASCT/HDT, distinguishing vaccine effects from delayed treatment response was challenging in this study because CR, PR and stable disease (SD) were already achieved in seven, six and one patients at onset of vaccination, post-ASCT/HDT (8). In one of the most recent studies published by Qazilbash et al., another strategy was applied in Id vaccination. In this randomized phase 2 trial, a prime-and-boost strategy was employed by collecting T lymphocytes from patients that had been vaccine primed *in vivo* with patient-specific Id-KLH, followed by ex vivo activation of the T cells with CD3/CD28 magnetic beads and reinfusing these cells after HDT and autologous hematopoietic stem cell transplantation (AHCT) with subsequent booster doses of the assigned vaccine. Even though the vaccine led to robust immune response which persisted up to +180 days post–AHCT, there was no clinical benefit in the group that was vaccinated with Id-KLH compared to the control group with only KLH (17). **Table 1** shows the summary of trials using Id protein/peptide- or DNA-based Id vaccine.

**Table 1:** Summary of trials using non-DC-based Id vaccines in MM.

Number of patients	Disease stage	Prior treatment	Vaccine platform	Vaccine protocol	Key clinical result	Ref/Year
n=8 (n=5 MM and n=3 healthy individual)	stage I-II	4 untreated and 1 chemotherapy	Auto protein IgG in alum	Immunized: 0, 2, 6 weeks. 3 patients received extra vaccines: 3–6 months (IC/SC)	All patients remained asymptomatic with no progression during the study. Disease progressed in 2 patients three months after study closure	(10)/1996
n=5	stage IIA	Two untreated and the rest chemotherapy or local radiotherapy	Purified serum M-component in alum solution	Vaccine at day 1 (ID), concurrent SC GM-CSF daily (days 1-4). Repeat at 2, 4, 6, 8, and 14 weeks	At 1 year follow-up post-vaccination, one patient showed >50% reduction in M-component concentration; others remained stable	(15)/1998
n=12 (1 patient failed to finish due to PD)	Stage II-III	HDT followed by PBSCT	Auto Id conjugated to KLH	Id-KLH conjugates at 0, 2, 6, 10, 14, 24, 28 weeks (SC). IL-2 (2 patients) or GM-CSF (10 patients) near vaccine site for 5 days (SC)	FFDP ranged 9-36 months from first Id/KLH injection to first treatment after vaccination or last follow-up	(12)/1999
n=15	Stage II-III	First remission after HDT and PBSCT	Auto Id/KLH conjugates	Id/KLH at 0, 2, 6, 10, 14, 24, 28 weeks (SC). GM-CSF for 5 days (SC)	Id/KLH did not eliminate residual tumor. Median PFS: 40 months; OS: 82 months. Retrospective case-matched analysis found similar results with IFN-alpha alone or with steroids	(16)/2004
n=28	Slowly progressive stage I (n = 20) or asymptomatic stage II disease (n = 8)	Untreated (n = 24) or in a stable unmaintained response/plateau phase (>1 year; n = 4) following chemotherapy or radiotherapy	Monoclonal IgG and alum	Vaccination over 110 weeks (ID) with IL-12 (n = 15) or IL-12 and GM-CSF (n = 13)	Id immunization with GM-CSF and IL-12 induced T-cell responses more frequently than with IL-12 alone. The immune response correlated with a prolonged median time to progression (108 weeks, range 29 to 371+) for responders (n = 16) compared to non-responders (26 weeks, range 4 to 330+)	(14)/2007
n=15	Advanced MM	HDT, HSCT and various therapies such as PI, IMiD, chemotherapy, corticosteroids	Purified paraproteins linked to phage	Six vaccine doses at day 1, 7, 14, and weeks 4, 8, 12 (ID), with GM-CSF adjuvant (SC for three days post-vaccination) or KLH control as antigen control	Subset (80%, middle dose) showed clinical response, paraprotein levels decreased or stabilized	(13)/2014
n= 15 (1 removed due to PD)	Stage I, II	Chemotherapy and/or radiotherapy and/or thalidomide maintenance	DNA fusion vaccine, single chain variable fragment linked to fragment C of tetanus toxin	Vaccination at week 0, 1, 2, 4, 8 and 12 (IM)	Over the 52-week study period, serum paraprotein was undetectable, decreased or remained stable for ten patients, whilst ongoing CR/PR was maintained for 11. The median time to progression was 38.0 months for 13/14 patients. OS:64% after a median follow-up of 85.6 months	(8)/2015
n=36 (KLH, n = 20; Id-KLH, n = 16)	Stage I-III	Newly diagnosed who received induction therapy	Id conjugated with KLH or KLH and vaccine primed auto lymphocyte infusion	28 days pre-auto-HSCT: KLH or Id-KLH vaccines and GM-CSF. Auto lymphocytes day +2 to +5 post-HSCT. Additional arm-specific immunizations at days 30 and 90 after auto-HSCT	No difference in 3-year PFS between arms	(17)/2022

PBSCT, Peripheral blood stem cell transplantation; HSCT, Hematopoietic stem cell transplantation; Auto, Autologus; FFDP, Freedom from disease progression; GM-CSF, Granulocyte-macrophage colony-stimulating factor; HDT, High-dose chemotherapy; IC, Intracutaneous; ID, Intradermal; IM, Intramuscular; IMiD, Immunomodulatory drugs; KLH, keyhole limpet hemocyanin; OS, Overall survival; PD, Disease progression; PFS, Progression free survival; PI, Proteasome inhibitor; SC, Subcutaneous; CR, Complete response; PR, Partial Response.

### 2.2 DC-based Id vaccines

The professional APCs, DCs, play a critical role in the initiation and regulation of innate and adaptive immune responses. By exploiting their ability to potentiate host effector and memory CD8 T cell responses critical for anti-tumor immunity, DC vaccines have emerged as one of the leading strategies for cancer immunotherapy (18). DC-based vaccines were first tested in context of Id antigen in MM. Historically, Id DC-based vaccines have been tested in over a decade in MM trials with no clear clinical benefit. Several optimization methods have been proposed for the generation, route of administration, and timing of vaccination. The original methodology involved the isolation of DCs from peripheral blood harvested by leukapheresis and subsequent density gradient centrifugations (19, 20). Subsequently, the addition of cytokines, namely GM-CSF and IL-4, became standard practice for culturing either adherent mononuclear peripheral blood cells (21, 22), CD34+ (23) or CD14+ cells (24, 25). Various maturation protocols were suggested following Id-pulsing to enhance effectiveness, including exposure to tumor-necrosis factor alpha (TNF-α) alone (26, 27), TNF-α and IL1-B (28), CD40 ligand (29) or a combination of TNF-α, IL-6, IL-1, and prostaglandin E2 (30).

Different vaccination routes, including subcutaneous (23, 28), intradermal (24, 26), intravenous (20, 22, 27), and intranodal (29) have been investigated. In a phase I/II trial, Curti et al. compared Id-pulsed DCs through sequential subcutaneous and intravenous routes. Each patient served as their own control, undergoing sequential subcutaneous and intravenous administrations. Results showed that subcutaneous administration induced a more robust T-cell response, suggesting its effectiveness in eliciting immunological responses compared to intravenous infusion (25). Additionally, the vaccines were tested in diverse patient groups, encompassing those with advanced myeloma (21, 23, 31), following chemotherapy (23, 31), individuals with no pre-treatment (30), those with lower tumor burdens following ASCT (19, 27) and smoldering MM (SMM) (29). As mentioned, despite extensive efforts, the Id-DC based vaccines did not yield satisfactory clinical outcomes.

A study in 1998 by Wen et al. is one of the first trials of a DC-based Id vaccine against MM, and involved a 43-year-old patient with advanced-stage refractory myeloma. DCs were isolated from the mononuclear cells by a series of density gradient centrifugations. Although the clinical response was limited, this study confirmed the functional abilities of ex-vivo-generated myeloma DCs by producing Id-specific immune responses (31). Later some optimization was applied to the DC generation by culturing the adherent mononuclear cell with cytokines such as GM-CSF and IL-4. In 1999, two patients with advanced refractory MM received an autologous DC vaccine loaded with Id antigen and KLH, accompanied by GM-CSF as an adjuvant. The results showed that both patients developed an Id-specific T-cell proliferative response, characterized by the production of interferon gamma (IFN-γ). However, there was no clinical benefit in these two cases (21). Nevertheless, considering that these patients had advanced disease, it was anticipated that the vaccination might not exert an optimal effect on this particular group. Therefore, additional trials have been conducted on cohorts of patients with less compromised immune systems or lower tumor burdens, with the anticipation that this might yield enhanced efficacy of the vaccine. Six myeloma patients with early-stage/early-relapse were vaccinated with autologous DCs (intravenously) prepared as in the previous study and loaded with Id antigens and KLH. However, only a period of stable Id serum levels was observed for 3 patients and 2 patients experienced disease progression post-vaccination (22). In another study, DC-based Id vaccination was tested following HDT and peripheral blood progenitor cell transplantation (PBPCT) in 26 patients where lower tumor burden was expected. Notably, only four patients developed an Id-specific proliferative T cell response. Three of these immune responders were in CR at the time of vaccination. A total of 17 patients were alive at a median follow-up of 30 months after transplantation. These findings suggested that a lower tumor burden might lead to better immune responses. However, attributing these outcomes solely to the vaccine was challenging due to the absence of a control arm for comparison (20). Investigation of DC-based Id vaccination was also performed in untreated stage-I patients. In this trial, nine patients received autologous monocyte-derived DCs pulsed with Id and KLH. The vaccine led to T-cell proliferation in 56% of patients. As for other studies, the clinical response was modest, with slight reductions in M protein observed in three patients (30).

Efforts to enhance efficacy of DC-based Id vaccination have led to optimizations in DC generation. In early studies with DC-based vaccination, immature DCs were pulsed with the antigen. However, immature DCs have suboptimal ability to activate T cells. Therefore, DCs exposed to antigens in the presence of maturation-inducing cytokines was introduced. This was first tested in five patients in stable partial remission after HDT. Furthermore, it was proposed to switch the administration route from intravenous to subcutaneous to overcome potential limitations linked to intravenous vaccination such as the potential accumulation of DCs in organs like the lung, liver, and spleen. Even with modifications to DC generation and a shift in the administration route, the clinical benefits were not remarkable. Moreover, attributing the clinical response solely to DC vaccination remained challenging, especially given the timing initiated four months after chemotherapy/auto-HCT (28).

Despite exploring optimizations in DC generation, administration routes, vaccination timing, and addition of immunogenic carrier protein/adjuvants, no remarkable results were observed. Bendandi et al. hypothesized that the lack of clinical significance could be explained by a deficiency in both quantity and quality of DC obtained from MM patients. Therefore, they performed a pilot study in which 4 MM patients received allogeneic dendritic cells (alloDC) followed by Id-KLH. The vaccine only induced detectable anti-KLH effector T- and B-cell responses without inducing T-cell proliferation against tumor-specific Id. From a clinical standpoint the results were not impressive with one patient with SD after stopping vaccination, while 3 of them progressed within study follow-up (24). **Table 2** shows the summary of trials using DC-based Id vaccine in MM.

**Table 2:** Summary of trials using DC-based Id vaccine in MM.

Number of patients	Disease stage	Prior treatment	Vaccine platform	Vaccine protocol	Clinical result	Ref/Year
n = 1	Advanced-stage refractory myeloma	Chemotherapy	DC loaded with Id	3 IV doses at a 2-week interval	Poor clinical response. Id dropped after the first vaccine, but increased again 5 weeks after the first dose despite two additional doses	(31)/1998
n=12	Stage III	HDT and PBSCT	Auto DC-based Id vaccine/Id-KLH conjugate	2 IV infusions of Id-pulsed DCs + 5 SC boosts of Id/KLH. Vaccine received 3-7 months after HDT	9/12 remained alive post-auto transplantation (minimum 16+ months follow-up), 2 died and 1 patient succumbed to acute leukemia	(19)/1999
n= 2	Advanced refractory myeloma	Chemotherapeutic regimes and auto-HSCT	Auto DC pulsed with Id and KLH	4 doses every 2 weeks, followed by SC GM-CSF	Rise of paraprotein was slowed in one patient but progressed 1 month following last vaccination and there was no change in paraprotein in another patient	(21)/1999
n= 6	Early-stage/early-relapse MM	3/6 patients had chemotherapy and 3/6 no previous chemotherapy	Auto DCs loaded with Id and/or KLH	n=5 got 3 IV doses each, and n=1 received 2 IV vaccines	1 showed a minor (25%), persistent serum Id reduction. Stable Id serum levels in 3, while 2 had PD post-vaccination. 1 patient couldn’t be evaluated (died of infection)	(22)/1999
n = 11	Stage II and III	Chemotherapy	Auto DCs loaded with Id	1 SC vaccine dose, followed by 3 boosts of Id peptide either with GM-CSF (9/11) or with Id-loaded DCs (2/11)	Three months post-vaccination, one patient was staged as SD, Nine patients had PD evaluated 8 weeks after the application of the DC	(23)/2000
n = 26	MM clinical stage IIA	HDT and PBPCT	Auto DCs loaded either with Id or with Id-KLH conjugates	12 patients got 2 doses Id-loaded DCs (IV); 14 got 2 doses DCs loaded with Id/KLH (IV). Patients received 5 SC boosts of Id-KLH at 4-week intervals. Vaccine received 3-9 months after PBPCT	A total of 17 patients were alive at a median follow-up of 30 months after transplantation	(20)/2000
n = 5	Stable partial remission	HDT and auto-HSCT	Auto DCs were loaded with Id	3 doses at 2-week intervals (SC/IV), followed by SC boosts of low-dose recombinant IL-2 for 5 days after each vaccination. Vaccine received 4-33 months post transplantation	50% reduction in serum M-component in one immunologically responding patient for 6 months and SD for 6 months in three other immunological responsive patients. The non-responsive patient relapsed	(28)/2002
n = 12 (8 received full doses)	Stage II and III	HDT and PBPCT	Auto DCs loaded with Id	2 IV doses at day 0 and day 15, followed by boosters: 5 SC Id/KLH booster immunizations (every 4 weeks) co-injected with GM-CSF for 3 consecutive days. Vaccination started 3-6 months following PBPCT	2 patients remained in clinical PR at 25 and 29 months post-vaccination; 10 progressed, 6 eventually died from disease or complications	(27)/2003
n = 4 (None of the patients received full dose)	Patients with relapse or progressing disease following RIC allogeneic HSCT	RIC allogeneic HSCT, and rescue therapy with donor lymphocyte infusion or chemotherapy	Allogeneic DCs loaded with Id and Id-KLH conjugate	Up to 3 cycles, each with 3 vaccinations (ID). First cycle monthly, second bimonthly, and last every 3 months. Boosters: Each dose with SC Id-KLH conjugate, combined with GM-CSF	Clinically, results were modest: 2 progressed, 2 stopped vaccination after patients´ desire	(24)/2006
n = 15	Stage IA, IIA, IIIA	HDT, followed by tandem ASCT and maintenance therapy (IFN-α/Dex)	Auto DCs loaded with either VDJ-derived peptides or with whole Id-protein. KLH was always mixed with Id	Three SC and two IV DC injections at 2-week intervals, with possible monthly SC injections in case of SD based on DC availability. The median time from ASCT to vaccination was 48 months	7/15 had stable serological disease, 1 achieved PR, and 7 progressed	(25)/2007
n = 9	8 SMM and 1 MM (all stable and did not need treatment	No prior treatment (8/9) or auto-HSCT (1/9)	Auto DCs loaded with Id and KLH	4 vaccines on days 1, 14, 21, and 28 (IN), followed by SC IL-2 for 5 consecutive days after each DC vaccination	At 1-year follow-up, 6/9 had SD; 3 had slow progression during vaccination. At 5 years, 4/6 maintained SD	(29)/2010
n = 9	MM clinical stage I	5 patients had bisphosphonates, and 3 had localized radiation	Auto DCs were loaded with Id and KLH	5 doses at a 4-week interval (IV) (5/9) or SC (4/9)	M protein decreased slightly in 3 patients, while the remaining 6 showed slight to moderate increases	(30)/2011
n = 24, n=11: vaccinated arm and n=13: control group	stage I, II and III	1-4 previous lines of systemic therapy	Auto DC pulsed with Id	6 doses at a monthly interval (ID)	During the follow-up (median: 33.1 months), the disease remained stable in 7/11 (64%) of patients. Compared to 13-patient control group, no direct treatment effect observed. More vaccinated patients stayed in SD phase than the control group	(26)/2012

PBSCT, Peripheral blood stem cell transplantation; DC, Dendric cell; Auto, Autologus; DEX, Dexamethasone; GM-CSF, Granulocyte-macrophage colony-stimulating factor; HDT, High dose chemotherapy; HSCT, Hematopoietic stem-cell transplantation; Id, Idiotype; ID, Intradermal; IFN, Interferon; IN, Intranodal; IV, Intravenous; KLH, keyhole limpet hemocyanin; MM, Multiple myeloma; PD, Disease progression; PR, Partial response; RIC, Reduced intensity conditioning; SC, Subcutaneous; SD, Stable disease; SMM, Smoldering Multiple Myeloma.

## 3 Tumor-associated antigen

### 3.1 Peptide-based TAA vaccine

Although Id vaccination induces detectable immune responses, it fails to show clinical efficacy. This can possibly be attributed to the inhibitory effect of high levels of circulating Id proteins on T cells which leads to tolerance, and the fact that Id protein is a weak antigen (8, 29). Vaccines with TAA have been developed as an alternative to Id vaccines. Various TAAs are studied in MM both in clinical, pre-clinical and *in vitro* setting, such as Mucin-1 (MUC1), Receptor for hyaluronan-mediated motility (RHAMM), B-cell lymphoma 2 (BCL-2) family, Wilms tumor 1 (WT1), X-box binding protein 1 (XBP1), syndecan-1 (CD138), CS1 (SLAM7), cancer testis antigens (CTA), Dickkopf-1 (DKK1), methylmalonate-semialdehyde dehydrogenase (MMSA-1), heat shock protein (HSP), telomerase reverse transcriptase (hTERT) and survivin. **Table 3** shows the summary of trials using TAA as an antigen in MM.

**Table 3:** Summary of trials using tumor-associated antigen (TAA) as an antigen in MM.

Number of patients	Disease characteristics	Prior treatment	Antigen	Vaccine platform	Vaccine protocol	Key clinical result	Ref/Year
n=15	Residual or biochemically progressive disease following ASCT. Median time from last therapy to vaccination was 15 months (range: 3–134 months)	1-3 lines prior therapies	MUC1	Peptide-based	6 or 12 bi-weekly ImMucin vaccines (ID), co-administered with GM-CSF	Median time from first vaccination: 24 months (range 5.5–41.3); at which 10/15 patients had PD. Median PFS for the cohort approximately 17.5 ± 3.9 months	(32)/2015
n=10 (4 MM patients)	Stage IIIA MM	At least one standard therapy	RHAMM	Peptide-based vaccine emulsified with montanide ISA-51	RHAMM R3 peptide (300 μg) on day 3 as well as GM-CSF on days 1-5: 4 times SC at a biweekly interval	2 MM patients exhibited reduced plasma cells, β2-microglobulin, and decreased free light chains. 1 had PD and 1 had no change after vaccination	(33)/2008
n=9 (3 MM patients)	MM patients with PR or nCR after HDT and ASCT. Vaccine was administered 12 months after last chemotherapy	Chemotherapy	RHAMM	Peptide-based vaccine emulsified with montanide ISA-51	RHAMM R3 peptide (1000 μg) on day 3 as well as GM-CSF on days 1- 5: 4 times at a biweekly interval SC	One patient with MM showed a reduction of light chain in serum. 1 had PD and 1 had no change	(34)/2010
n=7 (4 received full dose, 1 dropped out after 2 vaccinations)	Relapsed MM patients	2-5 lines of therapy	Bcl-2, Bcl-XL and Mcl-1	Peptide-based vaccine mixed with montanide ISA-51	Vaccinated 8 times in 4 series of bortezomib treatment (vaccination day 2 and 9)	Out of the 6 evaluable patients, 3 showed signs of increased immune reactivity after vaccination. 2/3 of immune responders completed the vaccination protocol, 1 of whom had a PR and went on maintenance vaccinations before developing PD	(35)/2016
n=1	Chemotherapy-resistant MM	1. DMVM regimen, 2. cyclophosphamide and predonisolone	WT1	Peptide-based emulsified with montanide ISA 51	Weekly injections of HLA-A*2402-restricted 9-mer WT1 peptide for 12 weeks (ID)	Post-vaccination, there was a 60% decrease in BM cancer cells, a reduction in urine M protein levels from 3.6 to 0.6 g/day	(36)/2007
n=22 (12 vaccine monotherapy and 10 vaccine+len, one from the combination therapy discontinued)	SMM (moderate or high risk) of progression to MM		XBP1, CD138, and CS1	Peptide based emulsified in montanide ISA 720 VG	6 biweekly PVX-410 doses (SC) with concurrent IM poly-ICLC injections	When used alone, all patients had SD as their best response, with 5/12 progressed within 12 months. In the combination therapy group, one patient achieved a PR, and four had MR or SD each	(37)/2018
n=1	Stage IIIA	Chemotherapy with VAD, DCEP, CAD	MAGE-A3	Allogenic vaccine primed mononuclear cells and recombinant fusion protein comprising the full MAGE-A3 sequence, a portion of the H. influenzae ProtD reconstituted in the proprietary adjuvant AS02B	Syngeneic PBSCT and transfusion of vaccine primed peripheral blood mononuclear cells followed by repeated patient immunizations	The patient remained in remission 2.5 years after the second transplant	(38)/2007
n=27	Patients with measurable disease (based on serum/urine electrophoresis studies or serum-free light chain studies) or complete remission in case of high-risk cytogenetic features	Median of 2 prior lines of treatment (range 1–5) with lenalidomide, bortezomib-based therapy, or both	MAGE-A3	Vaccine primed auto T cells and MAGE-A3 multi-peptide vaccine (compound GL-0817) combined with Poly-ICLC, GM-CSF ± montanide ISA 51 VG	MAGE-A3 peptide immunizations were administered before T cells collection. T cells were transfused after ASCT followed by five additional booster vaccinations	2-year OS: 74%; 2-year EFS: 56%	(39)/2014
n=13	Symptomatic MM, who were within 12 months of starting treatment, had achieved ≥ VGPR. Vaccine was given at least three weeks after completing induction therapy	12/13 had lenalidomide and bortezomib during induction, and 9/13 had a change in induction therapy prior to enrollment	MAGE-A3	Fusion protein containing 109 amino acids of H. influenzae ProtD, the full-length MAGE-A3 protein, and a polyhistidine tail (His), and AS15. Vaccine primed auto lymphocytes	Pre-ASCT vaccination in conjunction with early post-ASCT vaccine-primed auto lymphocyte infusion. Immunizations #2-6 every three weeks from day 10 post-ASCT (days 10, 31, 52, 73, 94). Additional #7 and #8 given at 3-month intervals (IM)	Median PFS: 27 months; median OS not reached, showing no deviation from standard-of-care	(40)/2019
n= 54, arm A (n=28): PCV+ multi-peptide vaccine. Arm B (n = 26):only PCV	Symptomatic MM	1-4 prior therapies	TERT and survivin	PCV/Peptide emulsified in montanide ISA 51/Vaccine primed auto T cell infusion	TERT/survivin vaccine: SC injection. PCV: IM injection. GM-CSF: SC. Vaccine primed auto T cells at day 2 after transplantation followed by 3 vaccinations post-transplantation	In arm A, 36% developed immune responses to the tumor antigen vaccine, exceeding the study’s immunologic efficacy endpoint. However, this frequency did not translate into improved EFS compared to arm B	(41)/2011
n=12	Stage II or III. Vaccination started minimum 6 months after HSCT	Induction chemotherapy and HSCT with or without maintenance treatment	MAGE3, survivin and BCMA	TAA-mRNA-loaded DC vaccination pulsed with KLH	Three times (IV/ID) at biweekly intervals. Re-vaccinations allowed after 6 months in the absence of PD requiring therapy	At last follow-up (median 25 months, range 9–53 months post-first vaccination), 10 of 12 patients were alive. Among them, 5 had SD, and 5 had PD	(42)/2013
n=20 (10 vaccinated and 10 control)	Symptomatic MM, who were within 12 months of starting induction therapy, had achieved ≥ VGPR	Induction therapy and ASCT	CT7, MAGE-A3, and WT1	DC based vaccine loaded with antigens via electroporation	Priming on day +12 post-ASCT, boosters on days +30 and +90 (ID). Lenalidomide maintenance therapy started ∼3 months after ASCT	Although not powered to assess clinical efficacy, treatment responses favored the vaccine arm	(43)/2022
n=14 (13 included in the primary efficacy analysis)	Newly diagnosed MM not having achieved CR with induction	Induction therapy	Survivin	DC vaccine transduced with an adenoviral vector encoded with full-length survivin (Ad-S), with mutations neutralizing its anti-apoptotic function	7 to 30 days prior to stem cell collection and 20 to 34 days after ASCT (ID). Prevnar13 vaccine as positive control	After 4.2 years, 6/7 maintained disease-free status. Estimated four-year PFS at 71%, surpassing IFM 2009 trial historical data (50% at 4.1 years) for this patient population	(44)/2023

Auto, Autologus; ASCT, Autologus stem cell transplantation; BM, Bone marrow; CAD, Cyclophosphamide; adriamycin and dexamethasone; DCEP, Dexamethasone; cyclophosphamide; etoposide; and cisplatin; DMVM, Dexamethasone; melphalan; vincristine; EFS, Event free survival; GM-CSF, Granulocyte-macrophage colony-stimulating factor; H. influenza, Haemophilus influenza; HDT, High-dose chemotherapy; HSCT, Hematopoietic stem cell transplantation; ID, Intradermal; IM, Intramuscular; IN, Intranodal; IV, Intravenous; KLH, keyhole limpet hemocyanin; Len, lenalidomide; MM, Multiple myeloma; MR, Minimal response; nCR, near Complete response; OS, Overall response; PBSCT, Peripheral blood stem cell transplantation; PCV, Pneumococcal conjugate vaccine; PD, Disease progression; PFS, Progression free survival; PR, Partial response; ProtD, Protein D; SC, Subcutaneous; SD, Stable disease; SMM, Smoldering Multiple Myeloma; TAA, Tumor-associated antigen; VAD, Vincristine; doxorubicin and dexamethasone; HLA, Human leukocyte antigen; VGPR, Very good partial response; CR, Complete response.

The glycoprotein MUC1 is found in various cancers, including MM. ImMucin, a 21-mer synthetic long-peptide vaccine was tested in a phase I/II study in 15 myeloma patients. ImMucin induced significant T-cell responses and increased antibody titers against MUC1. However, clinical efficacy was suboptimal with median PFS of 17.5 ± 3.9 months (32).

RHAMM-derived peptide R3 was investigated in a phase I/II study as another potential immunogenic antigen for hematologic malignancies, including MM. The initial study with a 300 μg vaccine in 10 patients with positive HLA-A2, including 4 with MM, showed positive clinical and immunological responses. Among MM patients receiving the 300 μg vaccine, 3 out of 4 showed increased specific CD8+ T cells and 2/4 showed reductions in clonal markers. However, the limited sample size warrants caution in drawing definitive conclusions (33, 34).

BCL-2 family protein was investigated in a phase 1 trial as another target antigen in MM. Patients with relapsed MM received vaccinations with peptides from Bcl-2, Bcl-XL, and Mcl-1 mixed with montanide ISA-51 as an adjuvant together with bortezomib. Due to peptide HLA-restriction, patients received peptides according to their HLA-A-positivity, i.e., HLA-A1, HLA-A2 and/or HLA-A3. Among 7 patients, 3 demonstrated vaccination-induced peptide antigen-specific T-cell responses compared to the baseline (before vaccination). However, small sample size hindered a conclusive assessment of clinical efficacy (35). The WT1 gene, linked to childhood renal tumor Wilms tumor, plays dual roles as an oncogene and as a key component of certain normal cellular processes. It is highly expressed in hematopoietic malignancies and solid cancers, including myeloma, where its expression increases with disease progression (45, 46). Studies have identified various epitopes in WT1 that can elicit WT1-specific cytotoxic T lymphocyte (CTL) responses in a human HLA-restricted manner. Therefore, WT1 peptide-based immunotherapy could be an option for patients with malignant diseases [30, 33]. In a case study by Tsuboi et al., a 57-year-old chemotherapy-resistant MM patient received weekly intradermal vaccinations with HLA-A*2402-restricted 9-mer WT1 peptide and montanide ISA51 as an adjuvant. Post-vaccination, there was a 60% decrease in BM cancer cells, a reduction in urine M protein levels from 3.6 to 0.6 g/day. Additionally, there was a notable shift in C-X-C chemokine receptor type (CXCR)4 positive cells, suggesting effective migration of WT1-specific CTLs to the tumor site (36).

Multi-peptide vaccines have also been investigated to combat limitations of single antigen peptide-based vaccination, including potential resistance by antigenic escape and downregulation of target antigens. PVX-410 is a peptide-based vaccine, which consists of 4, 9-mer peptides from three different antigens including XBP1, CD138, and CS1. In one study, 22 moderate to high-risk SMM patients positive for HLA-A2 were administered with PVX-410 subcutaneously and an adjuvant, poly-ICLC, with or without lenalidomide. PVX-410 consistently generated specific, durable immune responses, particularly enhanced with lenalidomide. However, overall clinical responses were still modest. When used alone, all patients had SD as their best response, with 5/12 progressing within 12 months. In the combination therapy group, one patient achieved a PR, and four had minimal response or SD (37).

DKK1, MMSA-1, and HSP have been investigated as TAAs with potential for vaccine therapy against MM. DKK1, a Wingless-related integration site (Wnt)/β-catenin signaling inhibitor, induces peptide-specific CTLs that recognize and lyse myeloma cells with positive HLA-A*0201 (47). MMSA-1 is a membrane protein specifically expressed in MM cells. When combined with DKK1 in a vaccine, it enhanced CTL responses in HLA-A*0201-restricted manner, improved survival, and alleviated bone destruction in pre-clinical model (1). Tumor cell-derived HSPs, like gp96, have been explored as TAAs in myeloma in mice. Pooled HSPs, including gp96 from established murine myeloma cell lines, showed promise as an off-the-shelf vaccine effectively treating mice with large myeloma tumor burdens with HSP combined with anti-B7H1 or anti–IL-10 monoclonal antibodies (48). However, further studies are needed to confirm their efficacy in clinical setting.

Moreover, CTA antigens, initially discovered in melanoma patients are targets for immunotherapy due to their limited expression on normal tissues. A study by Andrade et al., indicated that the expression of more than six CTA antigens on myeloma cells holds prognostic value, being associated with shorter overall survival (OS) in MM patients (49, 50). Prominent CTA antigens like NY-ESO-1, LAGE-1, MAGE-A1, MAGE-A2, MAGE-A3, and MAGE-C1 (CT-7) have demonstrated the potential to stimulate T-cell responses, making them candidates for cancer immunotherapy (38). Among the CTA antigens, MAGE-A3 have been tested in a clinical setting. In a case study a healthy donor, who was the patient’s identical twin, was immunized with MAGE-A3 protein along with AS02B as an adjuvant. Following a syngeneic peripheral blood stem cell transplant, primed donor cells were transferred to the patient. Subsequently, the patient received additional immunizations with MAGE-A3, along with a second transfusion of peripheral bone marrow mononuclear cells. The MAGE-A3 immunizations were well-tolerated and resulted in the development of robust MAGE-A3-specific antibodies, CTLs, and T-helper responses in both twins. Interestingly, the CTL response targeted a previously unknown HLA-A*6801 binding MAGE-A3 peptide, which remained detectable in the patient more than a year after the last immunization. Multiple T-helper cellular responses were detected with the dominant response to an HLA-DR11 restricted MAGE-A3 epitope. Encouragingly, the patient remained in remission 2.5 years after the second transplant. This case study suggests the potential efficacy of this personalized immunotherapeutic approach for treating MAGE-A3-positive MM patients (38). In another phase II study, the safety and efficacy of ex vivo expanded autologous T cells primed *in vivo* using a MAGE-A3 multi-peptide vaccine combined with Poly-ICLC and GM-CSF were evaluated in twenty-seven myeloma patients. The vaccine includes two HLA-A2–restricted class I epitopes and a promiscuous class II epitope. Results showed that MAGE-A3–specific CD8 T cells were detected in 7 out of 8 assessable patients with HLA-A2+. Furthermore, vaccine-specific T cells capable of producing cytokines were generated in 19 out of 25 patients. The 2-year OS was 74% and the 2-year event-free survival (EFS) was 56% (39). A similar study was performed by Cohen et al. where recombinant MAGE-A3 with AS15 immunostimulant were administered to 13 MM subjects pre- and post-ASCT in conjunction with early post-SCT vaccine-primed autologous lymphocyte infusion. The combination immunotherapy resulted in high-titer humoral immunity and durable and robust, antigen-specific CD4^+^ T-cell responses in all subjects. However, median PFS was 27 months, and median OS was not reached, suggesting no differences from standard-of-care (40).

Other CTAs tested in pre-clinical settings have been suggested as possible immunotherapy targets in MM. Spontaneous NY-ESO-1 antibodies and specific CD8+ T cells have been detected *in vivo* in NY-ESO-1 positive MM cases (51, 52). In a pre-clinical model, a complex vaccine named NACH, based on NY-ESO-1-alum-CpG ODN-HH2 was investigated. The Alum-CpG ODN-HH2 combinational adjuvant used in the NACH vaccine is a novel immune adjuvant. The vaccine demonstrated promising anti-tumor effects and immunogenicity in prophylactic and therapeutic models of murine MM. The NACH vaccine inhibited tumor growth, leading to a significant extension of survival time in mice. In the therapeutic model, seven out of ten tumor-bearing mice in the NACH vaccine group survived up to day 90, while all mice in the control group died within 40 days (53). LAGE-1a, another CTA antigen, frequently expressed in MM patients, shares high mRNA sequence similarity with NY-ESO-1. In silico analysis identified seven peptides present in both LAGE-1a and NY-ESO-1, recognized by T lymphocytes in different tumors. Therefore, it was hypothesized that an anti-NY-ESO-1 vaccine could potentially benefit MM patients with tumors that express LAGE-1a but not NY-ESO-1 (54). MAGE-C1 (55, 56), SPAN-Xb (57, 58), and SP-17 (49, 59) are other CTA antigens capable of eliciting CTL responses, rendering them targets for vaccine-based immunotherapy MM.

Similar to Id vaccines, the practical incorporation of peptide-based tumor-TAA vaccines into clinical practice have not occurred. A study by Rapoport A.P et al. compared immunogenicity of the rate-limiting catalytic subunit of the telomerase complex called hTERT (60) and anti-apoptotic protein survivin as a target antigen vaccine to pneumococcal conjugate vaccine in 54 MM patients. Patients positive for HLA-A2 (including any A2 allele) were assigned to arm A where they received hTERT/survivin vaccine. The study suggested that although this multi-peptide tumor antigen vaccine had a higher immune response frequency than reported for idiotype vaccines, TAA still falls short of that induced by microbial vaccines. This suggests that achieving a more substantial immune response to cancer vaccines may be necessary for significant long-term clinical benefits (41).

### 3.2 DC-based TAA

DC-based vaccines using TAA associated antigens have also been assessed as alternatives to Id antigens. Twelve stage II or III myeloma patients with minimum PR after induction chemotherapy and HCT/ASCT were vaccinated. DCs were generated from adherent CD14+ cells loaded with MAGE3, survivin and BCMA via electroporation and were pulsed with or without KLH. Notably, all patients developed strong anti-KLH T-cell responses, which indicates immune recovery after high-dose melphalan, and in two patients, vaccine-specific T cells were detected in delayed-type hypersensitivity biopsies. At last follow-up, 10 of the 12 patients were alive at a median follow-up of 25 months (range 9–53 months) after the first vaccination. Of these patients, 5 had SD and 5 had progressive disease (42). In another study, mRNA electroporation of DCs was conducted, utilizing Langerhans DCs loaded with CT7, MAGE-A3, and WT1. These Langerhans-type DC, derived from CD34+ hematopoietic progenitor cells, were considered potentially more potent stimulators of CTLs against tumor antigens in comparison to monocyte-derived DCs *in vitro*. The patients were randomized to receive either the vaccine within 100 days after ASCT or were placed in the control group without the vaccine. While the study was not powered to assess clinical efficacy, treatment responses favored the arm receiving the vaccine (43).

In one recently published study, Locke and colleagues designed a DC-based vaccine targeting anti-apoptotic protein survivin in a phase 1 clinical trial. DCs were engineered via adenoviral vector to express a version full-length survivin (Ad-S) containing two mutations that neutralize the anti-apoptotic function of wild-type survivin. Thirteen newly diagnosed MM patients who did not achieve CR with induction therapy were vaccinated 7 to 30 days prior to stem cell collection and 20 to 34 days after ASCT. The vaccine in combination with ASCT was well tolerated, with only minor adverse effects noted. Notably, a remarkable 85% of patients exhibited either a T-cell response or an antibody response against survivin. Seven patients exhibited enhanced clinical responses at day +90, all linked to survivin-specific immune responses. Impressively, after a median follow-up of 4.2 years, six out of these seven patients maintained a disease-free status. The estimated four-year PFS was 71%, surpassing historical data from the IFM 2009 trial with approximately PFS of 50% at 4.1 years for this patient population (44). Survivin has emerged as a notable TAA in MM. Among the 8 ongoing trials in MM, two are focused on developing vaccines targeting survivin. One such vaccine is named TXSVN, utilizing a weakened form of a live Salmonella bacterial strain genetically modified to produce survivin. The second vaccine is a peptide-based formulation known as SVN53-67/M57-KLH, derived from the survivin protein (**Table 4**).

**Table 4:** List of ongoing vaccine trials against MM.

NCT Number	Study Title	Study Status	Conditions
**NCT03631043**	Personalized Vaccine in Treating Patients With Smoldering Multiple Myeloma	Not recruiting	SMM
**NCT02334865**	SVN53-67/M57-KLH Peptide Vaccine in Treating Patients With Newly Diagnosed Multiple Myeloma Receiving Lenalidomide Maintenance Therapy	Not recruiting	MM/Leukemia
**NCT02886065**	A Study of PVX-410, a Cancer Vaccine, and Citarinostat +/- Lenalidomide for Smoldering MM	Not recruiting	SMM
**NCT01067287**	Blockade of PD-1 in Conjunction With the Dendritic Cell/Myeloma Vaccines Following Stem Cell Transplantation	Not recruiting	MM
**NCT03762291**	Multiple Myeloma Trial of Orally Administered Salmonella Based Survivin Vaccine	Not recruiting	MM
**NCT05841550**	The TG01 Study With TG01/QS-21 Vaccine in Patients With High-risk Smouldering Multiple Myeloma and Multiple Myeloma	Recruiting	MM/SMM
**NCT03376477**	Allogeneic Myeloma GM-CSF Vaccine With Lenalidomide in Multiple Myeloma Patients in Complete or Near Complete Remission	Not recruiting	MM
**NCT06435910**	Engineered Dendritic Cell Vaccines for Multiple Myeloma	Recruiting	MM

Data is derived from https://clinicaltrials.gov. MM, Multiple Myeloma; SMM, Smoldering Multiple Myeloma.

## 4 Tumor-specific antigen

### 4.1 Peptide-based TSA vaccine

While certain studies on TAAs show promise, the overall assessment is challenging due to the limited patient numbers in each clinical trial. Further research with larger cohorts is necessary for conclusive findings on the efficacy of TAA-based immunotherapies. Moreover, due to their status as non-mutated self-antigens, in general TAAs may exhibit low immunogenicity attributed to the influence of central T cell tolerance. Therefore, TSAs, also called neoantigens, appear as another type of target antigens for vaccine immunotherapy (61, 62).

Somatic mutations in cancer cells can give rise to novel protein sequences that can be presented by APCs as neoantigens to the host immune system. Tumor neoantigens represent excellent targets for immunotherapy, due to their specific expression in cancer tissue (63). The landscape of neoantigens in 184 MM patients, as identified through next-generation sequencing, revealed shared neoantigens in NRAS, KRAS, and Interferon regulatory factor 4 (IRF4) genes in relapsed patients and in KRAS in newly diagnosed patients supporting the possibility of neoantigen-based vaccines in MM patients with such mutations (63). Perturbations in the MAPK pathway, particularly mutations in KRAS, NRAS, and BRAF, are observed in a significant percentage of MM cases, with RAS mutations detected in up to 70% of relapsed/refractory cases (64). These mutations, associated with MAPK activation, may impact prognosis, contributing to transitions from precursor conditions to myeloma and from intramedullary to extramedullary disease with increasing prevalence as the disease progresses (64). Targeting shared neoantigens, such as those associated with RAS mutations, is being explored in clinical trials, exemplified by the ongoing study by our group using the TG01 cancer vaccine in high-risk SMM or MM patients (**Table 4**) with measurable disease after ≥1 line of treatment. TG01, composed of 7 synthetic peptides mimicking mutated forms of the RAS protein, has demonstrated promising results in patients with pancreatic cancer. The primary endpoint of the TG01-study is safety, with key secondary endpoints including immunological response to the vaccine, overall response rate, OS and PFS (65).

Another neoantigen-based vaccine is PGV-001. PGV-001 is a personalized genomic vaccine which targets up to 10 predicted personal tumor neoantigens based on patient’s HLA profile. PGV-001 was tested in 13 patients with multiple cancer types in the adjuvant setting including three patients with MM who had undergone ASCT. Vaccine peptides were administered over the course of 27 weeks with poly-ICLC and a tetanus helper peptide. One patient was lost to follow-up, but among the remaining 12 patients with a mean follow-up of 925 days, four showed no evidence of disease, 4 received subsequent lines of therapy, and 4 have passed away. Notably, only two of the deceased patients had documented recurrence of their malignancy. The details of the patients and which of them were MM is not described in this abstract. Immune monitoring of immunogenicity is ongoing. However, initial analysis demonstrated induction of neoantigen-specific CD4 and CD8 T cell expansion (66).

As noted earlier, TSA prompt the activation of high-avidity T cells, given their lack of thymic selection and central tolerance. While there has been significant focus on Id, showing limited clinical efficacy in myeloma due to its abundance, research on other neoantigens than Ids in myeloma remains limited. Further studies are essential to evaluate their potential as target antigens in myeloma vaccine therapy (61).

## 5 Whole tumor antigen

### 5.1 DC-based vaccine

Single antigen-specific vaccines are vulnerable to immune evasion due to downregulation of antigen expression. Alternatively, whole cell targets have also been explored as a strategy, which seek to establish a polyclonal immunologic response. Such vaccines could potentially include a broad array of antigens, including neoantigens (2). This is particularly relevant in myeloma, where a significant tumor mutation burden exists. A study on 664 newly diagnosed myeloma patients found mean somatic and missense mutation loads of 405.84 and 63.90 mutations per patient, respectively. There was a positive relationship between mutation and neoantigen burdens (67). Additionally, high-dose melphalan therapy, a standard treatment before ASCT, is associated with a high mutational burden and possibly more neoantigens at relapse in myeloma patients (68).

Rosenblatt et al. developed a tumor vaccine by chemically fusing patient-derived myeloma cells with autologous DCs. This vaccine offered the advantage of potentially presenting a diverse range of myeloma-associated antigens within the context of DC-mediated co-stimulation. Fusion cells were created by co-culturing DCs and myeloma cells with polyethylene glycol. The DC fusion vaccine was tested in 17 patients, the majority of whom had advanced disease. Vaccination was well-tolerated and resulted in the expansion of lymphocytes that reacted with the patient’s own myeloma cells in most evaluated patients, as well as documented humoral responses. Patients with advanced disease experienced disease stabilization, with three patients showing ongoing SD at 12, 25, and 41 months, respectively. In the current study, regulatory T cell levels remained stable throughout the vaccination period (69). The same vaccine was further tested in patients following ASCT with the hypothesis that autologous transplantation provides an optimal setting for a fusion vaccine due to the enhanced immunologic environment resulting from tumor cytoreduction and regulatory T-cell depletion. The study achieved promising outcomes with 47% of patients achieving a CR/near CR (nCR) as best response and 78% of patients achieving at least a VGPR. Notably, nearly 35% of CRs occurred greater than 100 days post-transplant, after undergoing vaccination. Although delayed effects of chemotherapy may be observed, the significant number of late responses in the absence of maintenance therapy might be suggestive of a vaccine-mediated effect (70). Based on these results the group was encouraged to examine the efficacy of the fusion vaccine in conjunction with lenalidomide as maintenance therapy after auto-HCT. However, DC/MM fusion vaccination with lenalidomide did not result in a statistically significant increase in CR rates at 1-year post-transplant but was associated with a significant increase in circulating MM–reactive lymphocytes indicative of tumor-specific immunity (71).

The same group conducted another clinical trial involving MM patients who received an anti-programmed cell death protein 1 (PD-1) antibody (Pidilizumab) in combination with a DC/myeloma fusion cell vaccine following autologous transplantation with the aim to overcome the immunosuppressive milieu by which tumor cells evade host immunity. This trial involved 22 patients and showed that this combination could induce anti-tumor immune responses, and, in a subgroup of patients, led to the complete eradication of measurable disease after transplant. After the transplantation, regulatory T cell levels decreased significantly and remained low throughout the immunotherapy period. Six patients achieved a best response of VGPR, and six patients reached nCR/CR. The median PFS from the transplant was 19 months, with ongoing follow-up (**Table 4**) (72). Other whole cell lysates such as GVAX and DCOne vaccine has been tested as off-the shelf vaccines with a diverse antigen repertoire. MM-GVAX is a vaccine, in which two established heterogeneous myeloma cell lines, H929 and U266, are administered with the K562 cell line (GVAX^®^) engineered to overexpress GM-CSF. H929 exhibited t(4;14), and a mutated NRAS, and U266 has several mutations involving the BRAF and TP53 pathways. Patients enrolled were serum/urine immunofixation positive and maintained nCR for at least 4 months. In the publication from 2021, of 15 patients, 8 (53.3%) had deepened treatment response and achieved true CR. The median OS was 7.8 years. MM-GVAX triggered clonal T-cell expansion and cytokine responses that have remained durable up to 7 years in all patients. This trial is ongoing (**Table 4**) (73, 74). DCOne is another off-the shelf tumor vaccine, which is derived from a human acute myeloid leukemia (AML) cell line that can be differentiated to the phenotype of fully functional DCs, and endogenously expresses TAA. These antigens are presented alongside HLA molecules and various co-stimulatory molecules critical for T-cell activation. Although originally engineered as a cancer vaccine in AML, DCOne expresses tumor antigens found in a variety of hematologic malignancies, including MM. MM with the DCOne vaccine resulted in the expansion of activated CD8+ T cells expressing interferon-γ and perforin. Further, co-culture of patient’s tumor cells with peripheral blood mononuclear cells and DCOne induced cytotoxic T-lymphocyte-mediated killing of autologous MM cells, highlighting the therapeutic potential of DCOne in myeloma (2). **Table 5** shows the summary of trials using whole tumor antigen in MM.

**Table 5:** Summary of trials using whole tumor antigen in MM.

Number of patients	Disease characteristics	Prior treatment	Antigen	Vaccine platform	Vaccine protocol	Key clinical result	Ref/Year
n=18 (n=1 removed due to inadequate cell yields for vaccine generation)	Advanced disease/2 stage I with no prior treatment	Median of 4 prior treatment regimens (Range 0-6)	Auto plasma cell	DC/tumor fusions	3 doses with 3-week intervals (SC) with GM-CSF at the vaccine site	Most patients with advanced disease showed disease stabilization, with 3 displaying ongoing SD at 12, 25, and 41 months, respectively	(69)/2011
n=36 (Cohort 1: n=24, Cohort 2: n=12)		Median of two regimens (1–5)	Auto plasma cell	DC/tumor fusions	Both cohorts: 3 post-transplant vaccinations at 4-week intervals. Cohort 2 had an extra pre-mobilization vaccination. GM-CSF (SC) at the vaccine site days 1-3. Median time from transplant to vaccination was 1.3 months	47% of patients achieving a CR/nCR as best response and 78% of patients achieving at least a VGPR. Nearly 35% of CRs occurred greater than 100 days post-transplant, after undergoing vaccination	(70)/2013
n=22			Auto plasma cell	DC/tumor fusions	Patients received 3 doses of pidilizumab at 6-week intervals. DC/myeloma fusion cells vaccination was administered 1 week before each dose of pidilizumab. Median time from transplant to immunotherapy was 80 days	6 patients achieved VGPR, and 6 reached nCR/CR. Median PFS from transplant was 19 months, with ongoing follow-up	(72)/2015
DC/MM fusions with GM-CSF and lenalidomide (n=68), lenalidomide and GM-CSF (n=37), or lenalidomide alone (n=35)	Newly diagnosed MM	n= 14 received initial systemic therapy prior to enrollment	Auto plasma cell	DC/tumor fusions	All patients started lenalidomide maintenance ~3 months post–auto-HSCT. In cycles 2-4 of lenalidomide maintenance, vaccine arm patients received vaccine (SC) and GM-CSF adjacent to the vaccine site on days 1-4	Vaccination with lenalidomide did not result in a statistically significant increase in CR rates at 1 year post-transplant but was associated with a significant increase in circulating MM–reactive lymphocytes indicative of tumor-specific immunity	(71)/2023
n= 14 observational group, n=15 GVAX	Patients with sustained nCR for at least 4 months	Range of 1-4 prior treatments in both groups	Irradiated combination of H929 and U266 cells together with the K562 cell line (GVAX^®^)	MM-GVAX and PCV	MM-GVAX vaccinations (ID) at 1, 2, 3, and 6 months with lenalidomide at pre-enrollment dose + PCV (IM)	8/15 (53.3%) had deepened treatment response and achieved true CR. The median OS was 7.8 years from enrollment. MM-GVAX triggered clonal T-cell expansion and cytokine responses that have remained durable up to 7 years in all patients. This trial is ongoing	(74)/2021

Auto, Autologous; CR, Complete response; DC, Dendric cell; GM-CSF, Granulocyte-macrophage colony-stimulating factor; HSCT, Hematopoietic stem cell transplantation; ID, Intradermal; IM, Intramuscular; nCR, Near complete response; OS, Overall survival; PCV, Pneumococcal conjugate vaccine; PFS, Progression free survival; SC, Subcutaneous; SD, Stable disease; VGPR, Very good partial response.

## 6 Conclusion and future perspective

Despite extensive research, tumor vaccine has not yet been incorporated into myeloma therapy. The predominant emphasis has been on Id vaccines for many years which has yielded occasional immunological responses but with limited clinical efficacy. Subsequent investigations have explored diverse antigenic targets in clinical trials. Nevertheless, the widespread testing of various antigens in early-phase trials with restricted patient cohorts poses challenges in establishing definitive conclusions. Moreover, direct comparisons between studies even with the same antigen type are hindered by variations in patient characteristics such as age, cytogenetics/risk factors, and the disease stage at which vaccination occurred. Furthermore, a significant number of vaccine studies have been conducted during periods when chemotherapy constituted the standard of care and there was more focus on using vaccine alone to eradicate tumor. However, the combination of vaccination with newly established treatments like IMiDs, proteasome inhibitors, and new generation immunotherapies such as chimeric antigen receptor T (CAR-T) cells and bispecific antibodies has not yet been addressed.

**Figure 1:**
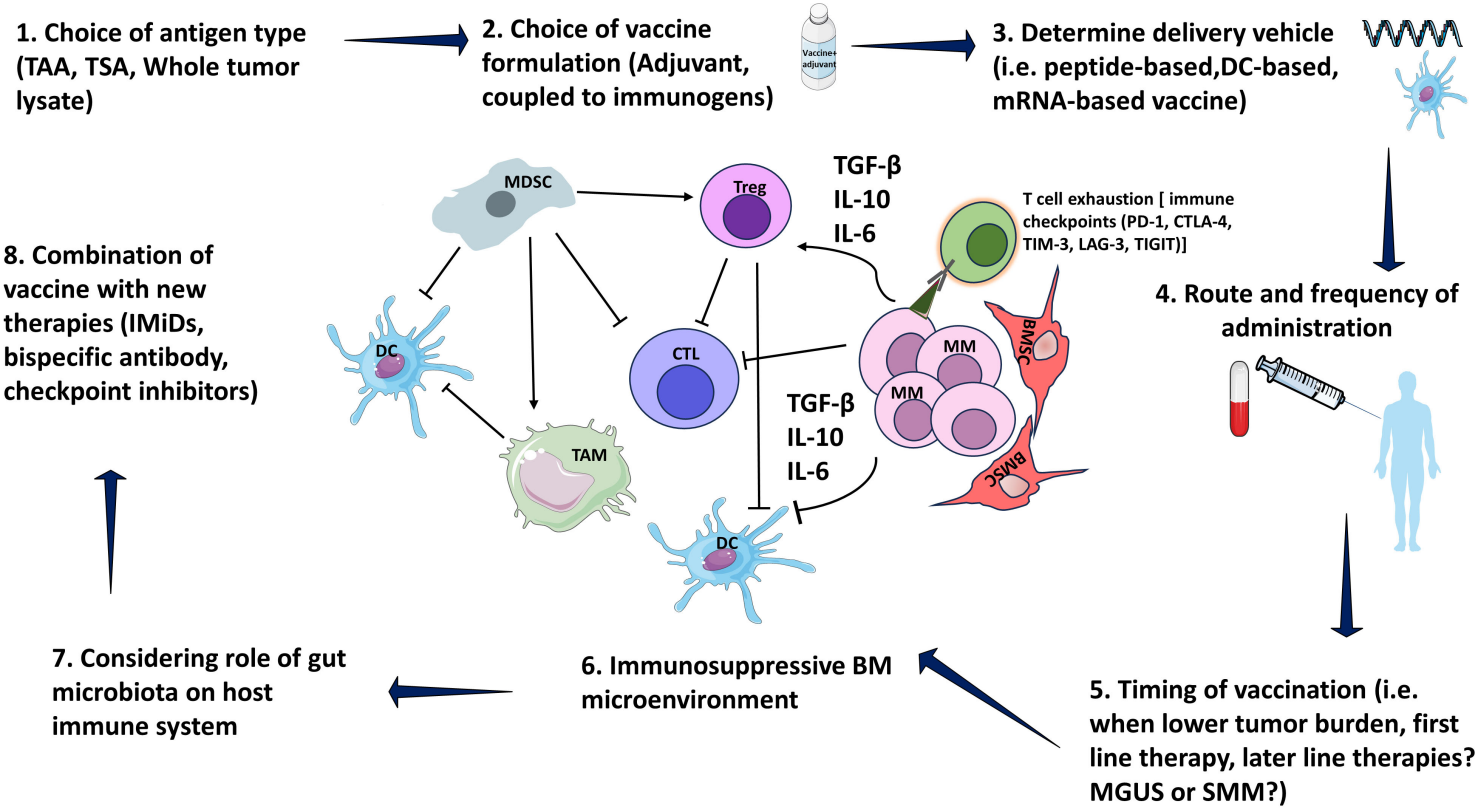
Consideration in designing vaccine. MM bone marrow microenvironment is consisted of immunosuppressive elements including MDSCs, TAM and Tregs. MDSCs stimulate TAM as well as Tregs via IL-10. MDSCs also inhibit CTLs via IL-10. Tregs inhibit CTL, and DC function by direct cellular interactions and via secretion of suppressive cytokines, such as TGF-β and IL-10. Moreover, MM cells secrete several cytokines including IL-6, TGF-β, and IL-10 that inhibit DCs, CTLs, and stimulate Tregs. Myeloma patients express multiple immune checkpoint receptors, including PD-1, CTLA-4, TIM-3, LAG-3, TIGIT. Terminal T cell exhaustion is associated with the loss of cytotoxicity by CD4+ and CD8+ T cells subsets that produce IFN-γ, a critical cytokine for tumor immunity. MM, Multiple myeloma; MGUS, Monoclonal gammopathy of undetermined significance; SMM, Smoldering MM; BM, Bone marrow; DC, Dendritic cells; BMSC, Bone marrow stromal cells; CTL, Cytotoxic T lymphocyte; MDSCs, myeloid-derived suppressor cells; TAM, Tumor-associated macrophages; Treg, Regulatory T-cells; PD-1, programmed cell death domain protein 1; CTLA-4, Cytotoxic T-lymphocyte-associated protein 4; TIM-3, T cell immunoglobulin mucin-3; LAG-3, lymphocyte activation gene 3; TIGIT, T cell immunoglobulin and ITIM domain; Interleukin (IL)-10; TGF-β1, transforming growth factor-β1; TAA, tumor-associated antigen; TSA, tumor-specific antigen. Parts of figures were used/adapted from pictures provided by Servier Medical Art (Servier; https://smart.servier.com/), licensed under a Creative Commons Attribution 4.0 Unported License (https://creativecommons.org/licenses/by/4.0/).

Generally, the intricate nature of vaccines coupled with the complexity of myeloma as a disease, presents challenges for integrating vaccines into myeloma treatment strategies. Vaccine therapy is complex and requires thorough experimentation. This includes selecting the tumor antigen, formulating the vaccine, determining the delivery vehicle (62), deciding on the route and frequency of administration, establishing the timing of vaccination in addition to accounting for the complexities of the tumor microenvironment in MM (**Figure 1**). Optimizing all these factors collectively may be necessary to improve clinical efficacy.

### 6.1 Antigen selection

Ideally, the target antigen must be immunogenic, highly expressed in tumor, low or absent in normal tissues and tumor-specific to avoid off-target effects (2). Several antigens, including Ids, TAAs, and TSAs, have been explored in myeloma research. Despite efforts to enhance their immunogenicity, especially for Ids by coupling vaccinations with immunogens and adjuvants, there has been no remarkable clinical efficacy (51). The study on TSAs other than Ids is still limited. Predicting TSA as targets is both time-consuming and costly, requiring advanced methods to predict neoantigens capable of eliciting neoantigen-specific T cell responses. Moreover, accurate HLA typing is crucial for reliably predicting immunogenic antigens and improving therapeutic efficacy, adding another layer of complexity to vaccine development. This is due to the polymorphism of human HLA alleles, which encompass over 24,000 unique gene complexes (6). Although there is strong rationale for the use of patient-specific vaccines that express multiple antigens such as whole tumor lysate, there is potential concern for the reestablishment of tumor tolerance over time facilitating disease progression (70). This could partly explain the delayed adoption of whole tumor lysate vaccine in myeloma clinical settings. Gut microbiota antigens possess inherent strong immunogenic properties, but remain an unexplored area in myeloma research. The presence of highly similar antigenic epitopes between TAAs and microbial antigens suggests the potential for a robust cross-reacting CD8+ T cell response. Specifically, T cell memory induced by specific microbial antigens may translate into anti-cancer T cell memory, exerting long-term control over cancer growth. Examples include molecular mimicry observed between MAGE-A10 and cytomegalovirus. This revelation opens avenues for exploring the cross-reactivity between myeloma-associated TAAs and microbial antigens, offering new insights for enhancing the efficacy of myeloma vaccine therapy (62).

### 6.2 Platform for vaccine delivery

Peptide-based vaccination has emerged as a promising method for eliciting antitumor T-cell responses. However, its effectiveness remains limited, even when the peptide is co-delivered with a potent adjuvant. This may be attributed to cancer-induced dysfunction in DC, which play an important role in influencing the quantity and quality of antitumor immunity. Consequently, the delivery of tumor antigens through DC-based peptide approaches is gaining recognition as a potential strategy in cancer immunotherapy (75). Since the inception of DC use in myeloma therapy, numerous studies have focused on optimizing DC generation, particularly utilizing idiotype as a source of antigens. Some studies have demonstrated the heightened potency of CD34+ hematopoietic progenitor cell (HPC)-derived Langerhans-type DCs to monocyte derived DCs in stimulating CTL (43). However, their clinical application in myeloma studies remains limited. In addition, there has been huge focus on ex-vivo generation of DCs derived from myeloma patients. Shinde et al. generated DCs from MM and healthy donor samples. While both MM-DCs and healthy donor-derived DCs showed mature phenotypes, MM-DCs exhibited lower migratory capacity and cytokine secretion, making them less effective in inducing an anti-MM response (76). Furthermore, efforts to enhance the delivery of antigens to DCs are being explored through various methods. These include pulsing DCs with antigen peptide pools and employing gene editing techniques such as electroporation (42), lipid nanoparticles (77), or viral vectors to improve antigen presentation (78). The COVID-19 pandemic led to notable advancements in mRNA vaccines, presenting an opportunity for their application in antitumor therapy. These vaccines offer robust cellular and humoral immunity, surpassing conventional pathogen or protein-based vaccines. Furthermore, mRNA vaccines offer advantages such as rapid development, safety, fewer side effects, and flexibility, thereby facilitating the implementation of personalized vaccine strategies, particularly in the context of TSAs. The utilization of mRNA vaccines for antigen delivery represents a novel approach warranting investigation for its potential in myeloma therapy (79, 80).

### 6.3 Route and timing of administration

The route of administration and the optimal duration of vaccination remain topics of ongoing debate, with insufficient comparisons in clinical trials. Another factor that has been overlooked in myeloma is the timing of tumor vaccine administration. In a large number of published clinical studies, vaccines are administered shortly after systemic anticancer treatment. This timing poses a challenge as natural immunity may be suppressed by chemotherapy at the time of vaccination (26). One suggested approach that has been explored in numerous trials involves the potential benefits of vaccination after ASCT (25, 43, 70–72), particularly when a state of deep response has been achieved. The engraftment of cellular immune compartments at this stage provides an opportunity to shape antitumor immunity. However, challenges to this approach include delayed immune reconstitution, with T cell numbers taking at least 3 months to return to normal, leading to suboptimal responses to vaccine therapy (40, 43). To address this issue, some studies have investigated pre-ASCT vaccination, followed by the early post-ASCT transfer of vaccine-primed T cells in a lymphodepleted environment. Post-transplant booster vaccinations were then administered to expand vaccine-specific T cells. Despite these efforts, this strategy did not yield favorable clinical outcomes (39–41). Nevertheless, these studies are limited, and more investigation is needed before drawing any conclusive results. Another potential approach is to target patients at an early stage in their disease course, particularly those with monoclonal gammopathy of undetermined significance (MGUS) or SMM. However, implementing this strategy would require treating a substantial number of patients and conducting long-term follow-up to establish clinical benefits. As of now, conclusive studies supporting this hypothesis are lacking (81). Three ongoing trials are currently investigating vaccines in patients with SMM, including our study called TG-01 targeting RAS mutation, PVX-410 (a multi-peptide cancer vaccine from XBP1, CD138, and CS1), and a personalized cancer vaccine made from an individual’s blood and bone marrow (**Table 4**).

### 6.4 Vaccine-extrinsic factors

Vaccine-extrinsic factors, including the tumor microenvironment and dysfunctional host immune responses in MM underscores the complexities of eliciting effective immune responses (43, 82). The MM tumor microenvironment is populated by immune-suppressive cells, including myeloid-derived suppressor cells, regulatory T cells, and tumor-associated macrophages. Additionally, inadequate antigen presentation, resistance to natural killer (NK) cell lysis, T-cell exhaustion and/or senescence, is associated with poorer outcomes after ASCT and characterizes multiple relapsed disease (43, 82). Myeloma-derived cytokines, like transforming growth factor (TGF)-β, IL-6 and IL-10, contribute to immune dysfunction (83, 84). In addition, vaccination effectiveness decreases with age due to T cell unresponsiveness resulting from age-related immune system changes (85). This is particularly significant for MM, with the average age at diagnosis being 70. Another extrinsic factor, which has been scarcely studied in myeloma patients, is the potential effect of gut microbiota on the efficacy of tumor vaccines. Radojević et al. reported a correlation between the composition of the gut microbiota and the immunogenicity of DCs by analyzing the fecal microbiota composition of 14 healthy donors, along with the phenotype and cytokines produced by monocyte-derived DCs [61]. Another study by Calcinotto et al. indicated that Prevotella heparinolytica promotes the differentiation of Th17 cells colonizing the gut and migrating to the BM of transgenic Vk*MYC mice, where they favor progression of MM (86). This subject is complex, as many factors can potentially affect gut microbiota including treatments like antibiotics which is commonly administered to myeloma patients.

### 6.5 Combination of vaccine with new generation therapies

It should be noted that most tumor vaccines alone do not directly eliminate tumor lesions, but tumor vaccines are potentially promising in eliminating MRD (62). Therefore, further trials are needed to define the role of vaccination in the era of new pharmacologic therapies such as immune checkpoint inhibitors (ICI), antibody-drug conjugates, bispecific antibodies, and CAR-T cells. Durable responses to these treatments are infrequent, but combination strategies with vaccines to prime anti-MM immunity offer an approach to boost responses or eliminate the MRD. For example, combination of vaccine with ICI seems reasonable approach but the safety concerns in the absence of a clear signal of improved efficacy have become a major obstacle to the clinical application of ICI in MM (43, 87). Vaccination prior to bispecific antibody constructs may augment therapeutic potency by creating pools of antigen-experienced T cells. Vaccines can also promote the expansion and efficacy of chimeric antigen receptor T cells. In addition, it should be noted that although bispecific antibodies and CAR-T currently dominate the MM immunotherapy landscape, cancer vaccines offer distinct advantages. Unlike CAR-T cells or bispecific antibodies, which primarily target cell surface tumor-specific antigens, cancer vaccines have the potential to target intracellular antigens. This capability presents an opportunity to address a broader range of tumor antigens (88).

### 6.6 Conclusion

In conclusion, even though vaccination against MM has so far not yielded very impressive clinical results, further studies focusing on selection of the appropriate antigen, patient population, optimization of timing and sequencing of vaccine, and identification of rational combinations are warranted. These elements, in combination with other immune-directed interventions to overcome the immunosuppressive activity of the tumor microenvironment may exert a greater benefit with improved and durable clinical responses.

## Author contributions

PA: Conceptualization, Writing – original draft, Writing – review & editing. HN: Conceptualization, Writing – original draft, Writing – review & editing. FS: Conceptualization, Writing – original draft, Writing – review & editing.
